# Evaluating the Distribution, Quality, and Educational Value of Videos Related to Shoulder Instability Exercises on the Social Media Platform TikTok

**DOI:** 10.5435/JAAOSGlobal-D-23-00034

**Published:** 2023-06-06

**Authors:** Mikhail A. Bethell, Albert T. Anastasio, Joshua R. Taylor, Troy Q. Tabarestani, Christopher S. Klifto, Oke Anakwenze

**Affiliations:** From the Department of Orthopaedic Surgery, Duke University Medical Center, Durham, NC.

## Abstract

Because of the lack of scientific oversight, the quality, applicability, and consistency of healthcare-related TikTok videos have become a focus of research exploration. The orthopaedic surgery literature lags behind other medical fields in analyzing the widespread utilization of TikTok videos for medical information delivery.

**Methods:**

TikTok was queried using the hashtag #shoulderstabilityexercises, and 109 videos were included. The videos were collected by two authors and independently evaluated using DISCERN (a well-validated informational analysis tool) and shoulder stability exercise education score (a self-designed tool for the evaluation of shoulder instability–related exercises).

**Results:**

DISCERN scores of videos uploaded by general users had significantly lower scores in all four categories than those uploaded by healthcare professionals (*P* < 0.001, *P* = 0.005, *P* = 0.002, and *P* < 0.001). For the shoulder stability exercise education score, general users had a significantly lower score than the healthcare professionals at 3.36 and 4.91 on a 25-point scale, respectively (*P* = 0.034). General users had more videos graded as very poor (84.2%) in comparison to the number of videos uploaded by healthcare professionals deemed very poor (51.5%). However, the remainder of healthcare professionals had their videos graded as poor (48.5%).

**Conclusion:**

Despite slightly improved video quality from healthcare professionals, the overall educational of the videos related to shoulder instability exercises was poor.

Given the range of motion of the shoulder in multiple planes, a complex interplay between dynamic and static restraints contributes to the stability of the shoulder.^1^ Shoulder instability occurs when structural elements fail to provide optimal tracking for the joint, leading to subluxation or dislocation events.^2^ With regard to etiology, Rowe et al determined that roughly 96% of shoulder dislocations were due to a traumatic event, and 4% were atraumatic due to repetitive use.^3^ Successful treatment of shoulder instability is dependent on accurate diagnosis, identification of existing structural defects, and recognition of abnormal movement patterns to inform design of a personalized rehabilitation program for each patient.^4^

Social media use as an avenue for consumption and distribution of healthcare information has steadily increased^5^ TikTok is a social media platform that was released for use in 2017. Users can share short mobile videos on various topics and can comment and like each video, leading to easier access within the platform's search algorithms. TikTok reaches a massive audience, and the platform had over 1.6 billion users and 2.6 billion downloads worldwide at the end of 2021.^6^ TikTok users have begun producing videos on various healthcare-related topics, and this trend was accelerated by the COVID-19 pandemic. Thus, the educational quality, applicability, and consistency with the literature and accepted medical opinion of healthcare-related TikTok videos have become a focus of research exploration. Topics of previous publication include COVID-19, cancer subtypes, diabetes, and chronic obstructive pulmonary disease, and TikTok videos have demonstrated variable quality and reliability across these analyses.^7,8,9,10^ Several authors have questioned the quality of the information presented on TikTok,^11,12,13^ and others have noted that healthcare-related posts on TikTok often contain no references and lack oversight from recognized health authorities.^7^ For example, Siegal et al^14^ and Jang et al^15^ determined that the quality of information on TikTok related to varicoceles and scoliosis exercises, respectively, was not acceptable for patient needs and relied on questionable sources.

The orthopaedic surgery literature lags behind other medical fields in acknowledging and analyzing the growing body of TikTok videos, which contain medical information. Because 55% of TikTok's userbase is between the ages of 18 and 24 years, TikTok may be an important source of easily accessible information for shoulder pathology relevant to younger patients, such as shoulder instability.^16^ Therefore, this study aims to assess the quality, reliability, and educational benefits of the information presented in videos on shoulder instability exercises on TikTok.

## Methods

### Search Strategy and Data Collection

**Figure 1:**
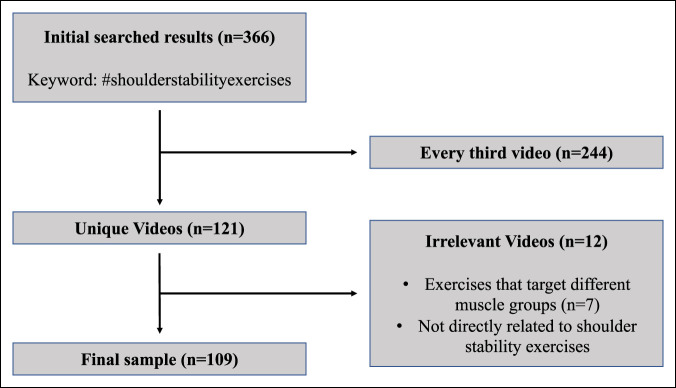
Flowchart of the search process for videos related to shoulder instability exercises.

The social media platform TikTok was queried to find videos related to shoulder stability exercises on August 15, 2022. The search was conducted by using the hashtag #shoulderstabilityexercises without any search filters. Thus, our intent was to analyze the videos that a TikTok user would be most likely to encounter if searching the platform for exercises related to shoulder instability. The search yielded a large number of total videos (n = 366). To add an element of randomization to the selection, we chose to include every third video for inclusion in the study (n = 121). Given that the search feature in TikTok does not list videos by any certain variable (i.e. highest viewed videos first), we elected to implement this pseudo-randomization method to account for differences in searches that would appear for the common platform user. On TikTok's official website, they acknowledge that the search results are partially determined by that user's interactions such as the videos they have previously liked or shared, accounts they follow, comments they posted, and content they may have already created.^17^ We then performed an initial screening of the videos and eliminated those that were (1) exercises that target different muscle groups (n = 7) and (2) not directly related to shoulder stability exercises (n = 5). After full screening, 109 videos remained after the data analysis (Figure 1). Previous studies evaluating video quality on TikTok for various medical topics have included lower video numbers and inadequately powered to detect important findings.^14,18,19^ Therefore, we aimed to include a higher number of videos.

For each of the videos included in the analysis, we recorded the creator of the video's data (username and video) and the number of views, likes, shares, comments, and favorites. We determined which uploaders were healthcare providers by looking for the presence of any advanced degree or specific licensing information on their TikTok profile. This study did not require any human participants or animals; thus, ethics committee approval was not required.

### Scoring System

Two separate scoring systems were used to evaluate the quality and educational value of the videos: the DISCERN, a previously validated tool used for evaluating the reliability and quality of a treatment approach, and the shoulder stability exercise education score (SSEES) to assess the education suitability of the information in each video.^20^ The SSEES was modified from a similar scale created for evaluating scoliosis exercise video quality from previously published work by Jang et al.^15^

#### DISCERN for Reliability and Quality Assessment

The DISCERN is a questionnaire, which provides researchers with a reliable and accurate way of assessing the quality of information on treatment choices for a health problem. The tool is well validated, has been used since the late 1990s, and is composed of 16 questions. The first set of eight questions assesses the reliability of the publication (DISCERN 1) and the next set of seven questions reviews the quality of the author's source base (DISCERN 2), and the final question then rates the publication as a whole in terms of its quality as a source of information (DISCERN 3).^20^ Although initially designed as a tool for written information, DISCERN has been successfully applied as a scoring test for grading the quality of videos in previous research.^21^ DISCERN scores are categorized as follows: excellent is denoted by scores of 63 to 75 points, good is denoted by scores of 51 to 62 points, fair is denoted by scores of 39 to 50 points, poor is denoted by scores of 27 to 38 points, and very poor is denoted by scores of 16 to 26 points.

#### Shoulder Stability Exercise Education Score for Educational Suitability Assessment

To grade the educational value of the videos, we developed the SSEES as a modification from previously published work by Jang et al.^15^ This test considers if the viewers can properly understand and follow rehab exercises after watching the video. The SSEES has five grading marks: “Exercise cycle (does the video describe the exercise cycle?),” “Target (does the video describe the target area of the exercise?),” “Effect (does the video describe the expected effect of the exercise?),” “Safety (does the video describe the precautions and safety components of the exercise?),” and “Rationale (does the video explain the rationale of the exercise?).” Each grading mark is scored between 0 and 5 with increasing scores demonstrating higher quality. The sum of all five grading marks is the final SSEES score (0 to 25, with scores of 0 representing the lowest possible quality and scores of 25 representing the highest possible quality).

#### Assessment

The videos were collected and independently evaluated by our orthopaedic research team. This team consisted of medical students and residents who were carefully trained by an attending surgeon to appropriately identify high-quality, evidence-based videos related to this topic. Once the data regarding video distribution metrics were collected for each video, the content of the videos was graded using the DISERN and SSEES tools. Each video was graded separately by two trained reviewers. Any points of discrepancy between the two reviewers were resolved by the senior author.

After scoring the videos, the uploaders were placed into groups based on the uploader's background into three categories: general users, healthcare providers, and health organizations. The healthcare provider category included users that described themselves as healthcare professionals such as chiropractors, physicians, physical therapists, and nurses. The health organization group includes clinics, hospitals, and treatment centers. In this study, there were no videos from users that were in the health organization group.

#### Statistical Analysis

Scoring and characteristic data are presented as the mean (standard deviation [SD]), median (interquartile range [IQR]), and percentage. For the basic characteristics of the videos, the data were reported using the median (IQR) rather than the mean to prevent outliers from skewing the results. A two-sample *t* test was used to compare the two types of uploaders by using the mean, SD, and sample size of each continuous and categorical variable. Statistical significance was set at *P* < 0.05 for comparisons. All analyses were performed using Microsoft Excel; (Redmond, Washington).

## Results

### Basic Characteristics

In total, 121 videos were pulled after searching #shoulderinstabilityexercises. Of the 121 videos, 12 videos were excluded after applying exclusion criteria and randomization protocol, and 109 videos were included in the final analysis. Table 1 presents the basic characteristics of the videos analyzed. The total number of views of the 109 videos included was 11,992,070, with a median of 4992 (IQR = 2264 to 21,400). The videos collectively received 761,472 likes, 4,210 comments, 152,786 favorites, and 35,121 shares with a median of 243 (IQR = 87 to 867), 5 (IQR = 1 to 18), 29 (IQR = 8 to 103), and 11 (IQR = 2 to 58), respectively.

**Table 1:** Characteristics of Included Videos

Characteristics, median (IQR)	Total videos included (n = 109)
Number of views	4992.0 (2264.0-21400.0)
Likes	243.0 (87.0-867.0)
Comments	5.0 (1.0-18.0)
Favorites	29.0 (8.0-103.0)
Shares	11.0 (2.0-58.0)
Scoring, mean (SD)	
DISCERN 1	13.60 (2.31)
DISCERN 2	9.07 (1.88)
DISCERN 3	2.00 (0.67)
Total DISCERN	24.63 (4.46)
SSEES	3.83 (3.52)

### Type of Uploaders

General users uploaded more videos (n = 76, 69.7%) when compared with healthcare professionals (n = 33, 30.3%). However, the characteristics between the general users and healthcare professionals were all statistically insignificant (Table 2).

**Table 2:** Characteristics of the Videos Across the Two Types of Uploaders

Characteristics, median (IQR)	General users (n = 76)	Healthcare professionals (n = 33)	*P*
Number of views	5577.0 (2737.8-28975.0)	4510.0 (1390.0-14700.0)	0.110
Likes	251.5 (112.0-1094.0)	242.0 (34.0-583.0)	0.120
Comments	5.0 (1.0-18.3)	3.0 (0.0-13.0)	0.146
Favorites	36.0 (10.8-107.8)	17.0 (5.0-59.0)	0.139
Shares	12.0 (3.0-55.0)	7.0 (2.0-59.0)	0.200
Scoring, mean (SD)			
DISCERN 1	12.98 (1.94)	15.03 (2.46)	<0.001
DISCERN 2	8.74 (1.55)	9.83 (2.33)	0.005
DISCERN 3	1.87 (0.54)	2.30 (0.84)	0.002
Total DISCERN	23.54 (3.62)	27.15 (5.19)	<0.001
SSEES	3.36 (2.92)	4.91 (4.49)	0.034

SSEES = shoulder stability exercise education score

The tabulated scores for the DISCERN 1, DISCERN 2, DISCERN 3, and total DISCERN were statistically significantly different (*P* < 0.001, *P* = 0.005, *P* = 0.002, and *P* < 0.001, respectively) between general users and healthcare professionals (Table 2). The DISCERN scores of videos uploaded by general users had significantly lower scores in all four categories than those uploaded by healthcare professionals (*P* < 0.001, *P* < 0.005, *P* < 0.002, and *P* < 0.001). For the SSEES, general users had a significantly lower score than the healthcare professionals at 3.36 and 4.91 on a 25-point scale, respectively (*P* = 0.034).

The DISCERN grading results are recorded in Table 3. General users had more videos graded as very poor (84.2%) in comparison to the number of videos uploaded by healthcare professionals deemed very poor (51.5%). Healthcare professionals had more videos graded as poor (48.5%) in comparison to the videos uploaded by general users (15.8%). However, none of the videos uploaded by either the general users or healthcare professionals were graded as fair, good, and excellent.

**Table 3:** Percentage of DISCERN Grades Across the Two Types of Uploaders

Grading	General users	Healthcare professionals	Total
Very poor	84.2%	51.5%	74.3%
Poor	15.8%	48.5%	25.7%
Fair	0%	0%	0%
Good	0%	0%	0%
Excellent	0%	0%	0%

## Discussion

Internet search engines and various medical informational delivery sites have provided patients additional means to obtain medical advice outside of the physician-patient interaction. The rise of various social media platforms has further expanded the availability of conveniently accessible information, which can be easily disseminated and shared^22,23^ Benefits of the current information age include improved access of medical information for patients in underserved areas and more involvement in patient-centered decision making. In recognition of these benefits, healthcare professionals have started to use social media to provide their knowledge with various communities and reach a wider audience outside of the patients they may interact with in person.^24^ However, the information age brings certain concerns, chiefly the potential for wide dissemination of biased, incomplete, or otherwise low-quality information on social media platforms.

Although the availability of the internet has certainly increased access to informational sources, health literacy and the ability to interpret this information have not necessarily improved in recent years. A documented lack of health literacy exists among adolescents and young adults in the United States, and poor health literacy has been found to be associated with adverse health outcomes^25,26^ Other work has shown that health literacy decreases with age and lower levels of education. Higher percentages of low health literacy are found in ethnic minority and economically disadvantaged communities.^27^ Despite the decrease in health literacy in younger age groups, younger individuals demonstrated more competency than their older counterparts in using Facebook and YouTube to find information related to various search topics.^28^ Thus, the emergence of TikTok and its enormous and rapidly expanding userbase of primarily younger members has allowed for the dissemination and consumption of a massive quantity of information. Previous investigation has shown that TikTok contains a variety of medical information pertaining to a vast number of topics such as COVID-19, chronic obstructive pulmonary disease, diabetes, and scoliosis.^9,10,15,29^ Although other specialties have evaluated the quality of the information pertaining to the common pathologies of their field, orthopaedics has been slow to recognize the rise in utilization of TikTok as an avenue for access to orthopaedic-related information. With almost 12 million total views of the included videos in our analysis (which represent roughly a third of all videos that are found with the search #shoulderstabilityexercises), TikTok reaches a massive audience and has the potential to profoundly affect patient perception of their pathology and treatment protocols.

Our data analysis revealed that videos of shoulder instability exercises on TikTok were found to be low in both quality and reliability. There were no videos that were categorized as fair, good, and excellent with an average DISCERN score of 24.63, with 74.3% of videos graded as very poor and 25.7% of videos graded as poor. Not only was the total DISCERN score low but the DISCERN 1, DISCERN 2, and DISCERN 3 were all tabulated as less than 50% of the maximum score for the DISCERN tool. In addition, the SSEESs were low, with the total average score under 25% of the maximal value for the SSEES, which indicates that the videos were deficient in describing components of the exercise, safety considerations, and the target rationale and effects. These deficiencies should be interpreted not necessarily as an indictment of the uploaders themselves but rather may be due to the confines of the TikTok platform. The short video playtime does not allow for appropriate citation of sources and robust discussion of risks and benefits. Moreover, TikTok and other video platforms may provide demonstrations of various exercises, but users do not have access to direct feedback when performing the exercises as would be available during a formal physical therapy visit. Repeated engagement in exercise with improper form may exacerbate instability by contributing to muscular imbalance.^30^

Our results are generally in concordance with previous studies evaluating the quality of TikTok videos for dissemination of healthcare information.^9,31,32^ Our study reveals worse scoring metrics than those reported by Jang et al., who found a total DISCERN score of 33.60 for the videos related to scoliosis exercises on TikTok. However, other studies have found TikTok to be a suitable outlet for the distribution of medical information for other fields outside of orthopaedics such as diabetes (total DISCERN score of 40 to 51),^9^ chronic obstructive pulmonary disease (total DISCERN score of 57 to 67),^10^ and medical abortion (modified DISCERN score of 2.8 of 5).^33^

Perhaps not surprisingly, this study found that the quality and reliability of shoulder instability exercises differed between general users and healthcare professionals. Despite general users having more views, likes, favorites, and shares than healthcare professional, they had lower scores for both the DISCERN and SSEES tools. Furthermore, although healthcare professionals did produce statistically higher quality videos than general users, the overall quality was still deemed to be poor and likely unacceptable for use as a stand-in for formal physical therapy purposes. TikTok lacks scientific oversight from recognized healthcare authorities to regulate the quality and completeness of healthcare information being posted. Moreover, proper citation of source material is rare: only one of the 109 videos included in the analysis provided a citation for the information contained within the video through inclusion of the PubMed ID.

## Limitations

There are several limitations in our assessment of the educational quality of the videos related to shoulder instability exercises posted to TikTok. The potential for selection bias exists with the search term used. We used a very general search term shoulderstabilityexercises to attempt to simulate a search term most users interested in this topic may use to obtain related videos. However, users who are familiar with their specific subtype of instability may favor more specific search terms. Furthermore, the process of grading videos contains inherent subjectivity in the assessment of the quality of the video. We attempted to mitigate this bias by including the use of a well-validated tool, the DISCERN, in addition to the use of the SSEES. To further address this issue, each video was independently reviewed by two separate reviewers, and a third reviewer was used where there was any significant discrepancy.

## Conclusion

Despite slightly improved video quality from healthcare professionals using the platform when compared with general users, overall educational value is poor. Providers should be aware of the high video counts and broad distribution of this material and should work to raise awareness of the deficiencies of the platform as a medium for educational medical-related information.
